# Lipid nitroalkene nanoparticles for the focal treatment of ischemia reperfusion

**DOI:** 10.7150/ntno.62351

**Published:** 2022-01-01

**Authors:** Gary Z Yu, Thiruganesh Ramasamy, Marco Fazzari, Xucai Chen, Bruce Freeman, John J Pacella

**Affiliations:** 1 Center for Ultrasound Molecular Imaging and Therapeutics, Department of Medicine, University of Pittsburgh, Pittsburgh, PA, USA;; 2 Department of Pharmacology and Chemical Biology, University of Pittsburgh, Pittsburgh, PA, USA.

**Keywords:** ultrasound-targeted lipid nanoparticle cavitation, microvascular obstruction, acute myocardial infarction, inflammation, nitro-fatty acid, nitroalkene

## Abstract

**Rationale:** The treatment of microvascular obstruction (MVO) using ultrasound-targeted LNP cavitation (UTC) therapy mechanically relieves the physical obstruction in the microcirculation but does not specifically target the associated inflammatory milieu. Electrophilic fatty acid nitroalkene derivatives (nitro-fatty acids), that display pleiotropic anti-inflammatory signaling and transcriptional regulatory actions, offer strong therapeutic potential but lack a means of rapid targeted delivery. The objective of this study was to develop nitro-fatty acid-containing lipid nanoparticles (LNP) that retain the mechanical efficacy of standard LNP and can rapidly target delivery of a tissue-protective payload that reduces inflammation and improves vascular function following ischemia-reperfusion.

**Methods:** The stability and acoustic behavior of nitro-fatty acid LNP (NO_2_-FA-LNP) were characterized by HPLC-MS/MS and ultra-high-speed microscopy. The LNP were then used in a rat hindlimb model of ischemia-reperfusion injury with ultrasound-targeted cavitation.

**Results:** Intravenous administration of NO_2_-FA-LNP followed by ultrasound-targeted LNP cavitation (UTC) in both healthy rat hindlimb and following ischemia-reperfusion injury showed enhanced NO_2_-FA tissue delivery and microvascular perfusion. In addition, vascular inflammatory mediator expression and lipid peroxidation were decreased in tissues following ischemia-reperfusion revealed NO_2_-FA-LNP protected against inflammatory injury.

**Conclusions**: Vascular targeting of NO_2_-FA-LNP with UTC offers a rapid method of focal anti-inflammatory therapy at sites of ischemia-reperfusion injury.

## Introduction

Cardiovascular disease is a leading cause of death in the United States, with over 1 million Americans having a new or recurrent acute myocardial infarction (AMI) in 2020 1. Contemporary treatment for AMI is percutaneous coronary intervention (PCI), which aims to restore perfusion to the myocardium through recanalization of the epicardial vessels for maximal myocardial salvage 2. Although mortality from AMI has decreased in recent years, post-MI congestive heart failure is increasing, due to subsequent microvascular obstruction (MVO) and inadequate restoration of microvascular perfusion, ultimately limiting myocardial salvage 3.

MVO is a phenomenon in which myocardial tissue remains hypo-perfused after PCI, even in the face of patent upstream epicardial vessels. This is due to a combination of distal micro-embolization, ischemia-reperfusion injury, activation of inflammatory cascades and tissue edema that contribute to vessel compression and local vasospasm 4-10. MVO occurs in up to 60% of all STEMI patients receiving PCI, thus decreasing LV function. MVO is an independent predictor for major adverse cardiac events, which include cardiac death, stroke, myocardial infarction, and heart failure requiring hospitalization 11-13. MVO is also associated with increased infarct size and LV remodeling, with persistence of MVO being a stronger predictor for functional recovery than transmural infarct extension 13-17.

Previous strategies for limiting MVO include vasodilatory and antiplatelet drugs, thrombus aspiration, embolic protection devices and hyperoxic intracoronary reperfusion therapy 18. No definitive therapeutic consensus for these various strategies for MVO has arisen, with the multiple clinical trials addressing these options yielding conflicting results 4, 6, 12. Therefore, there is an unmet need for an acute therapy capable of resolving MVO and its inflammatory sequelae due to the unpredictable and rapidly catastrophic nature of acute cardiac events. In addition, the combination of inflammatory and oxidative stress induced vascular dysfunction includes not only cardiac contexts, but is relevant to other vascular pathologies including diabetes, peripheral vascular disease, and reperfusion injury to the vasculature of many organs 19-21.

This unmet need is being addressed by the development and targeted delivery of anti-inflammatory electrophilic nitro-fatty acids (NO_2_-FA). These endogenous signaling mediators are generated by metabolic and inflammatory reactions of nitric oxide (NO) and nitrite (NO_2_^-^)-derived species with unsaturated fatty acids 22. These small molecule electrophiles target specific reactive cysteines (Cys) present in the redox-sensitive proteome, undergoing a reversible and transient Michael addition-mediated protein post-translational modification (PTM). These selective PTM of hyperreactive Cys moieties in turn induce downstream adaptive signaling and gene expression responses 23-26. Diverse gene expression studies and murine models of cardiovascular injury affirm the pleiotropic engagement of anti-inflammatory signaling mechanisms by NO_2_-FA 27-34. The net effects are decreased production of reactive species, inhibition of pathogenic M1 to M2 macrophage phenotype activation, inhibition of fibrotic and hyperplastic processes and reversal of endothelial dysfunction 35-39. All of these responses are relevant to the prevention and treatment of MVO, especially as systemically administered NO_2_-FA show cardioprotective effects in the setting of focal and global cardiac ischemic injury^29^, ^40^-^42^. In addition, NO_2_-OA inhibits xanthine oxidoreductase, a key pro-inflammatory source of reactive oxygen species in the vascular compartment 43, 44. This protective effect has been demonstrated previously in a murine model of hypoxia-induced pulmonary hypertension as well 45.

Notably, the targeted focal delivery of NO_2_-FA provides an immediate elevation in concentration of active drug at specific tissue sites, a novel and untested strategy for improving the pharmacokinetics of a covalent-modifier drug class 46. Given the relatively rapid onset of signaling effects and a lack of adverse responses to 10-nitro-octadec-9-enoic acid administration in preclinical pharmacokinetics and toxicology studies and human Phase 1 and 2 trials, we envisioned that NO_2_-FA entrapped in lipid nanoparticles (LNP), their intravenous injection and subsequent targeted ultrasound (US)-induced intravascular release represents a viable potential therapy for MVO.

Therapeutic ultrasound-induced thrombolysis is a growing subject of investigation, with ultrasound-targeted cavitation (UTC) of drug-free LNP shown to improve tissue perfusion following MVO 47, 48. This technique involves image-guided US-induced sonication of intravenously injected LNP contrast agents, resulting in LNP oscillation (cavitation) and the generation of intravascular shear forces. UTC produces both mechanical and biological effects by disrupting microthrombi, restoring perfusion, activating endothelial nitric oxide (NO) signaling and improving local vasodilation 48-50. In addition to thrombolysis, UTC can be utilized for targeted delivery of therapeutics via drug-loaded LNP, resulting in site-specific concentration of the therapeutic agent, thus limiting off-target effects 51-55. UTC is an ideal solution for treating MVO because it is a minimally invasive, theranostic technique that can visualize regions of MVO using LNP as US contrast agents, while simultaneously delivering image-guided ultrasound pulses.

We report herein a LNP formulation that contains a significant fraction of intercalated electrophilic NO_2_-FA (termed NO_2_-FA-LNP). These LNP were designed to a) augment the therapeutic efficacy of UTC for MVO, b) improve NO_2_-FA pharmacokinetics, c) facilitate the targeted delivery of NO_2_-FA, d) promote microvascular perfusion, e) induce anti-inflammatory responses and f) improve vascular protection. Herein, the physical properties, acoustic behavior and vascular effects in a rat hindlimb ischemia-reperfusion injury model reveal the therapeutic potential of NO_2_-FA-LNP. The rat hindlimb model mimics the microvascular structure of the heart and allows for US contrast-enhanced microvascular perfusion imaging in both a healthy state and upon ischemia-reperfusion injury. The results of this study indicated that NO_2_-FA-LNP treatment significantly limited oxidative and cytokine-associated inflammatory responses and improved hemodynamic parameters.

## Methods

### Nitro-fatty acid LNP synthesis and characterization

Nitro-fatty acid LNP were synthesized with a combination of 1,2-distearoyl-sn-glycero-3-phosphocholine, 1,2-Distearoyl-sn-Glycero-3-phosphoethanolamine-N-(methoxy(polyethylene glycol)-2000) (Avanti Polar Lipids Inc., Alabaster, AL), polyoxyethylene-40 stearate (Sigma Aldrich, St. Louis, MO), and 10-nitro-octadec-9-enoic acid (10-NO_2_-OA, NO_2_-FA). All components were combined in a glass vial with chloroform in a 29.1:5.2:43.8:21.9 mol ratio before the resulting solution was vaporized under an argon gas stream before vacuum storage. The dehydrated lipids were then rehydrated in acidic saline with a head of perfluorobutane gas (FluoroMed, LP, Round Rock, TX, USA) prior to sonication (XL2020, Qsonica LCC, Neton, CT). The resulting NO_2_-FA-LNP were then washed 3 times in acidic saline to remove unsonicated lipids from solution before being aliquoted into sealed glass vials containing perfluorobutane.

The NO_2_-FA-LNP were characterized for LNP concentration, size distribution, and loading efficiency of NO_2_-FA. All measurements were taken serially over 6 d post-synthesis to allow for tracking of stability in storage and NO_2_-FA content over time. Concentration and size distribution were measured using a Coulter Counter (Multisizer 3 Beckman Coulter, Fullerton, CA, USA). Structural characterization and quantitative analysis of 10-NO_2_-OA was performed by HPLC-ESI-MS/MS, using an analytical C18 Luna column (2 x 20 mm, 5 μm; Phenomenex) with a flow rate of 0.7 mL/min and a gradient solvent system of water 0.1% acetic acid (solvent A) and acetonitrile 0.1% acetic acid (solvent B). The gradient program was the following: 35-100% solvent B (0-3min) and 100% solvent B (3-4min) followed by 1 min re-equilibration to initial conditions. Detection of 10-NO_2_-OA was performed in negative mode using a QTRAP 6500+ triple quadrupole mass spectrometer (Sciex, Framingham, MA) with the following parameters: declustering potential -50 V, collision energy -42 eV, source temperature of 650°C, and a multiple reaction monitoring (MRM) transition 326.2/46. Quantification of 10-NO_2_-OA was performed by stable isotopic dilution analysis using a calibration curve using [^15^N]O_2_-[d_4_]OA as an internal standard (MRM 331.2/47). Free NO_2_-FA was subtracted from the overall solution NO_2_-FA measurement resulting in a concentration of only NO_2_-FA loaded onto NO_2_-FA-LNP.

Control LNP were synthesized using the same lipid components in the absence of NO_2_-FA and using the same procedures for quantification of size and concentration. For contrast-enhanced US imaging, DEFINITY® (Lantheus Medical Imaging, North Billerica, MA) was used.

### Analysis of NO_2_-FA in NO_2_-FA-LNP and tissue samples

For samples of NO_2_-FA-LNP, both the LNP in saline solution and samples of the saline subnatant alone were diluted in acetonitrile, spiked with 1.5 pmol [^15^N]O_2_-[d_4_]OA internal standard and measured for NO_2_-FA content by HPLC-MS/MS analysis. Subnatant NO_2_-FA concentration was subtracted from overall LNP solution NO_2_-FA concentration to give the concentration of incorporated NO_2_-FA only. For tissue samples, skeletal muscle tissue was taken from the treatment site (gastrocnemius muscle) and a control site (quadriceps femoris muscle) on the ipsilateral hindlimb, frozen in liquid nitrogen, and stored at - 80 °C. Frozen tissue samples (~200 mg) were thawed and homogenized with a bullet blender (Next Advance) in 50 mM phosphate buffer pH 7.4. An aliquot of the homogenate (500 μl) was spiked with 1 pmol [^15^N]O_2_-[d_4_]OA internal standard and extraction of NO_2_-FA was performed by adding 1.25 ml hexane- isopropanol-formic acid 1M (30:20:2, vol/vol/vol), vortexing and adding 1.25 ml hexane. After vortexing and centrifugation, the upper layer containing the NO_2_-FA was recovered, dried under nitrogen, and resuspended into 100 μl acetonitrile before HPLC-MS/MS analysis. The differential content of NO_2_-FA between the two sites was obtained by subtraction of respective NO_2_-FA concentrations.

### High speed microscopy

High-speed microscopic imaging was used to visualize the dynamic behavior of the NO_2_-FA-LNP during ultrasound excitation using a microscopy system capable of recording up to 25 million frames-per-sec for 128 frames.

### Ischemia-reperfusion injury model

Animal studies were approved by the Institutional Animal Care and Use Committee at the University of Pittsburgh (protocol number 18042556). A rat hindlimb model permitted the study of both healthy microvasculature and microvasculature experiencing ischemia-reperfusion injury in the same rodent 56. Anesthesia was induced using 2.5% inhaled isoflurane in 2-month-old male Sprague-Dawley rats (Charles Rivers, Pittsburgh, PA, USA) weighing 275 ± 20 g. The right femoral artery and right internal jugular vein were then cannulated for intravascular access. The distal right femoral artery was ligated, and the infusion line was advanced to the level of the iliac bifurcation for direct access to the left femoral artery. The jugular access was used to delivery DEFINITY^®^ for contrast-enhanced US imaging undiluted at a rate of 2 mL/hr. The left femoral access was used to deliver LNP +/- therapeutic agents. Treatment groups for the experiments included NO_2_-FA alone with a vehicle of 85:15 polyethylene glycol to ethanol (Free NO_2_-FA), UTC using unmodified treatment LNP with co-infusion of free NO_2_-FA (Free NO_2_-FA+UTC), and UTC with NO_2_-FA-LNP infusion only (NO_2_-FA-LNP+UTC).

### Ultrasound targeted cavitation therapy and perfusion imaging

For the UTC treatment, therapeutic US was administered using a single-element transducer operating at 1 MHz frequency (A303S, 0.5 inch, Olympus NDT, Waltham, MA), driven with an arbitrary function generator (AFG3252, Tektronix, Beaverton, OR) and a radio frequency power amplifier (800A3B, Amplifier Research, Souderton, PA). The ultrasound system was calibrated with a 200-µm capsule hydrophone (HGL-0200, Onda Corp, Sunnyvale, CA, USA). The treatment transducer was fixed vertically above the gastrocnemius region of the left rat hindlimb with the rat placed in right lateral decubitus position at a distance of approximately 1.5 cm from the hindlimb and was coupled to the hindlimb using acoustic gel.

The therapeutic US pulse (center frequency 1 MHz, peak negative acoustic pressure 1.5 MPa, pulse duration 5 ms) was delivered with 3 sec pulse interval to allow new LNP to reach the treatment area for 10 min for each treatment session. Each treatment session was followed by contrast-enhanced US imaging using a clinical imaging probe (Siemens, Sequoia, 15L8 probe) and infusion of DEFINITY^®^. LNP were infused with automated syringe pumps and the syringes were rotated during administration to maintain LNP in suspension.

Contrast-enhanced US imaging was performed using the burst-replenishment technique in contrast pulse sequence mode at 7 MHz frequency with a mechanical index of 0.2 and a frame rate of 5 frames/sec. The imaging US transducer was positioned horizontally, parallel to the longitudinal axis of the rat tibia and perpendicular to the treatment transducer. The imaging transducer was manipulated such that the junction of the saphenous and popliteal arteries was visible in the field of view and the skin thickness was minimized. Occasional artifacts potentially representing bone reflections were permitted but ultimately excluded from any regions of interest for analysis. A 5-frame burst with mechanical index of 1.9 was used to extinguish contrast LNP from the field of view. Both the initial burst and subsequent reperfusion of the hindlimb with contrast LNP were recorded for further processing and analysis of hemodynamic parameters. All other US parameters including dynamic range (60 dB), gain (0 dB), and compression curve (linear) were kept constant throughout the studies for all treatment groups.

### Image processing

Processing of all recorded cine-loops from burst-replenishment imaging was performed using in-house code in MATLAB as previously 56. Microvasculature regions of interest were selected for the gastrocnemius microvasculature such that skin, bone, and major vessels (saphenous and popliteal) were excluded. Image brightness (representing 60 dB range) of the region of interest was analyzed as a function of time for each cine-loop (approx. 30 sec) and fit to an exponential regression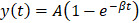, where 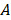 is the plateau value 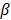 is the inverse time constant. The product 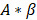 is the initial slope of the curve (accounting for the steepness of the increase of the brightness to the plateau value over time). Hemodynamically, the plateau value 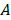 was taken to represent the microvascular blood volume while the product 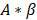 was taken to represent the microvascular blood flow rate.

### Healthy hindlimb model experimental protocol

Treatment groups for the healthy hindlimb model were centered on the analysis of tissue delivery of the loaded NO_2_-FA and included free NO_2_-FA alone, UTC of NO_2_-FA with co-infusion of unmodified LNP, and UTC of NO_2_-FA-LNP. The dosage of NO_2_-FA in all treatment groups was constant and represented the amount of NO_2_-FA content of 1×10^9^ NO_2_-FA-LNP. Similarly, all LNP dosing (regardless of NO_2_-FA loading) were 1×10^9^ LNP per treatment. All treatment infusions (NO_2_-FA or LNP infusions) were administered via the femoral cannulation at 3 mL/hr. All imaging LNP infusions were administered via the internal jugular cannulation at 2 mL/hr.

Placement of the therapeutic transducer over the gastrocnemius muscle was confirmed by visual inspection and one pulse of LNP destruction in the field of view prior to treatment. After each 10-min treatment, infusion of imaging LNP was performed during burst-replenishment imaging and cine-loop recording. Rat physiologic parameters including heart rate, respiratory rate, and oxygen saturation were continuously monitored. After the sec 10-min treatment, the rat was euthanized by isoflurane overdose followed by heart excision. Following euthanasia, tissue samples were obtained from the UTC-treated gastrocnemius muscle tissue as guided by US imaging markers. Tissue samples were also collected from the ipsilateral quadriceps femoris muscle as a control for the UTC treatment receiving the same systemic perfusion of infused agents without targeted US stimulation. All tissue samples were then immediately frozen in liquid nitrogen and stored at -80°C.

### Ischemia-reperfusion injury model experimental protocol

Aside from the induction of ischemia-reperfusion injury, the protocol for the rat hindlimb model was identical to that of the healthy hindlimb model. Prior to treatment, the left femoral artery was ligated using a suture, resulting in isolated left hindlimb ischemia. After 1 hr ischemia, the suture was removed, followed by 30 min reperfusion of the hindlimb. Then, the treatment proceeded as with the healthy hindlimb model. After the sec 10 min treatment and perfusion imaging, there was a 2 hr interval to allow for development of pathophysiologic changes in gene expression before animal euthanasia and tissue sample collection.

### Inflammatory response analysis

Malondialdehyde (MDA), measured as a thiobarbituric acid reactive substance (TBARS), is a product of enzymatic and non-enzymatic unsaturated fatty acid oxidation. Skeletal muscle tissue samples were homogenized in ice-cold buffer and centrifuged at 12,000 g for 15 min at 4°C. After centrifugation, supernatants were processed for TBAR products at 532 nm. TBA reacts with MDA to form a TBA product detectable at 532 nm (ε = 1.56 × 10^5^ M^-1^cm^-1^). Results are expressed per milligram of tissue protein measured using a standard Bradford assay (Thermo Fisher Scientific, Waltham, MA, USA).

The skeletal muscle tissue samples from the treatment and control groups previously described were homogenized in the presence of TRIzol reagent (ThermoFischer Scientific, Waltham, MA, USA) using an Ultrasonic Processor (Heat Systems, XL-2020). Chloroform (200 µL) was added to 1 mL of TRIzol solution, centrifuged at 12,000 g for 15 min at 4°C, and 200 µL of aqueous upper layer was pipetted into an RNAse-free centrifuge tube. After subsequent treatment with isopropyl alcohol and 75% ethanol, the RNA pellet was dissolved in DEPC water. Complementary DNA was prepared from 2 μg of total RNA using TaqMan reverse transcription kit (Applied Biosystems, Foster City, CA, USA). RT-PCR amplifications were performed with the SYBR™ Green PCR Master Mix (Thermo Scientific, Waltham, MA, USA) using an CFX96 Connect™ Real-Time PCR Detection System (Bio-Rad, USA). Target genes included intercellular adhesion molecule 1 (ICAM-1), vascular cell adhesion molecule 1 (VCAM-1), monocyte chemotactic protein 1 (MCP-1), tumor necrosis factor alpha (TNF-α), interleukin-6 (IL-6), and nuclear factor kappa-light-chain-enhancer of activated B cells (NF-κB). The relative mRNA levels of target genes were normalized to GAPDH mRNA. The primer sequence used for RT-PCR analysis includes:

TNF-α (For) 5'-CTCTTCTCATTCCCGCTCGT-3',

TNF-α (Rev) 5'-GGGAGCCCATTTGGGAACTT-3';

IL-6 (For) 5'-CCAGTTGCCTTCTTGGGACT-3',

IL-6 (Rev) 5'-TCTGACAGTGCATCATCGCT-3';

ICAM-1 (For) 5'-CAAACGGGAGATGAATGGT-3',

ICAM-1 (Rev) 5'-TCTGGCGGTAATAGGTGTAAA-3';

MCP-1 (For) 5'-CTGACCCCAATAAGGAATG-3',

MCP-1 (Rev) 5'-TGAGGTGGTTGTGGAAAAGA-3';

VCAM-1 (For) 5'-TTTGCAAGAAAAGCCAACATGAAAG-3',

VCAM-1 (Rev) 5'-TCTCCAACAGTTCAGACGTTAGC-3';

NF-κB (For) 5'-ACGATCTGTTTCCCCTCATCT-3';

NF-κB (Rev) 5'-TGGGTGCGTCTTAGTGGTATC-3';

GAPDH (For), 5'-AAACCCATCACCATCTTCCA-3',

GAPDH (Rev), 5'-GTGGTTCACACCCATCACAA-3'.

### Statistical analysis

All statistical analyses were performed in Prism (GraphPad Prism version 8.00, GraphPad Software, La Jolla, California, USA). Analyses were performed as either one-way ANOVA or two-way repeated measures ANOVA when appropriate. Post-hoc analyses for two-way repeated measures ANOVA were performed using Sidak's multiple comparisons test, while post-hoc for one-way ANOVA used Tukey's multiple comparisons test. Significance was taken at *p*<0.05. Error bars indicate standard deviation (SD).

## Results

### Nitro-fatty acid-lipid nanoparticle characterization

**Figure 1:**
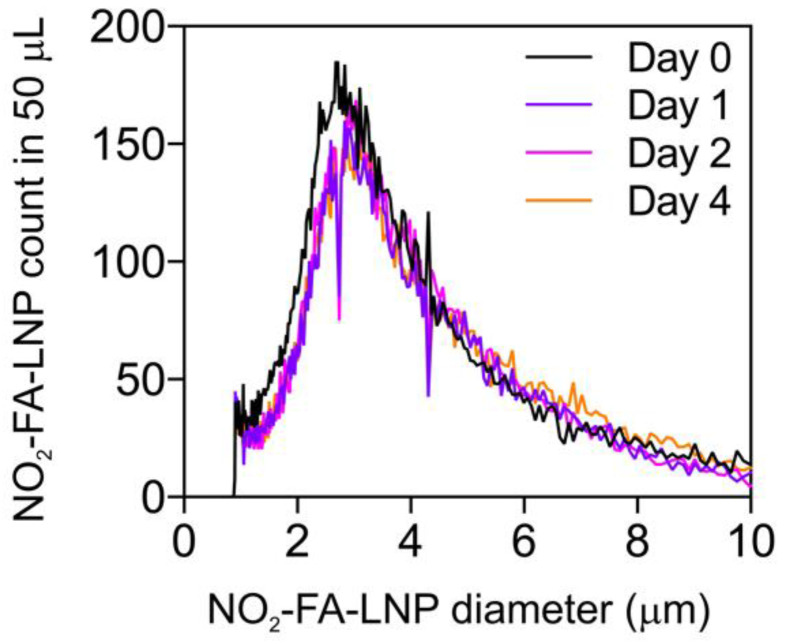
Size distribution of NO_2_-FA-LNP was stable over time - Coulter counter measurement of NO_2_-FA-LNP size and concentration distributions over time - Samples of NO_2_-FA-LNP from the same synthesis batch were serially sampled for measurement in a Coulter counter for distributions of LNP counts over LNP diameters, showing stability of the size and size distribution up to 4 d post-synthesis.

The size and size distribution of NO_2_-FA-LNP were measured by Coulter counter analysis (Fig. 1) for up to 4 d post-synthesis. There were no significant differences in NO_2_-FA-LNP diameter (3.0 ± 1.5 µm) over 4 d of storage, with mean NO_2_-FA-LNP concentration dropping ~15% from d 1 onward. Size distribution curves depict a unimodal population similar to that of previous LNP measurements 57.

**Figure 2:**
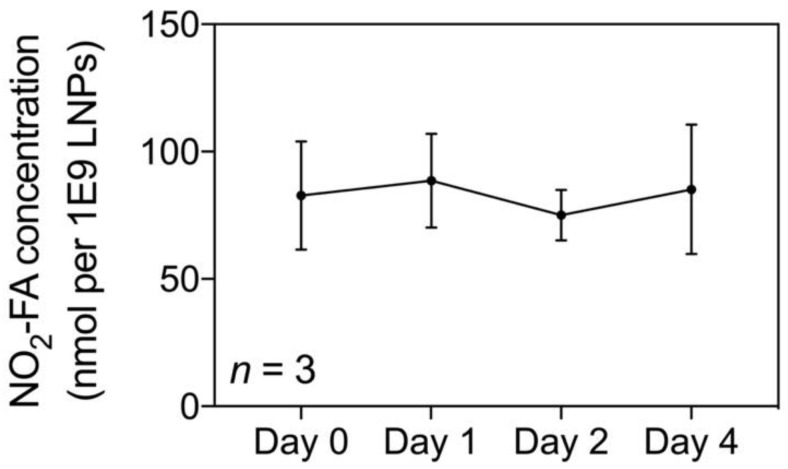
Quantification of loaded NO_2_-FA in NO_2_-FA-LNP - NO_2_-FA concentrations in saline were quantified by HPLC-MS/MS analysis. Subnatant samples with no NO_2_-FA-LNP were also quantified for concentration of free, unincorporated NO_2_-FA which was then subtracted from total NO_2_-FA concentration, resulting in the shown measurements. Three separate batches of NO_2_-FA-LNP were measured in triplicate for the presented data.

The NO_2_-FA content of NO_2_-FA-LNP was serially measured up to 4 d post-preparation (Fig. 2). The average concentration was 82.9 ± 5.8 nmol NO_2_-FA per 1×10^9^ LNP after subtraction of the subnatant containing NO_2_-FA that were not LNP-associated. Because the therapeutic dose for LNP previously established in the rat hindlimb model is 1×10^9^ LNP/mL delivered at 3 mL/hr, the NO_2_-FA content present in 1×10^9^ LNP (given a total treatment time of 20 min) was also used as the dosage present in free NO_2_-FA and free NO_2_-FA+UTC groups 58.

**Figure 3:**
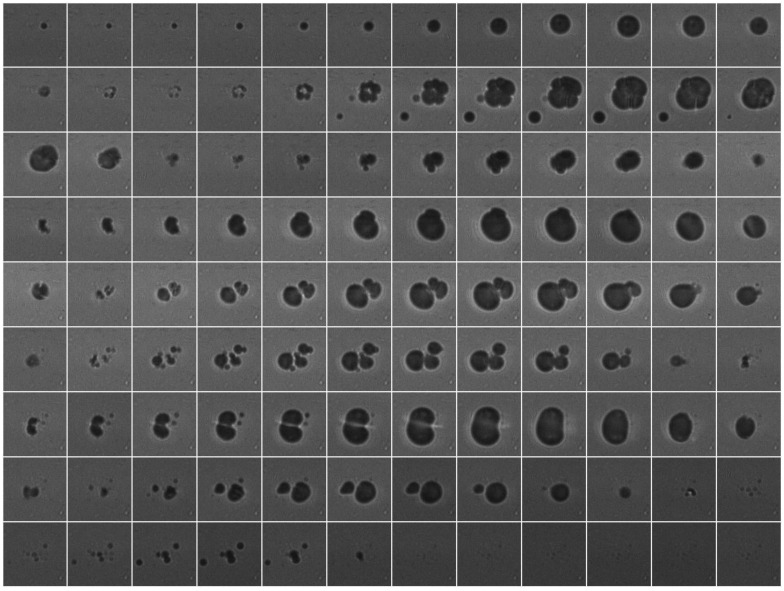
Sequential frames of high-speed video of NO_2_-FA-LNP stimulated by US (1 MHz, 1.5 MPa) show typical lipid LNP behaviors, including collapse and formation of daughter LNP. Frame size shown is 40 μm.

High-speed microscopy during US stimulation of NO_2_-FA-LNP revealed that NO_2_-FA-LNP behaved similarly as control LNP. Consecutive still frame images of a high-speed movie are shown in Fig. 3 (1 MHz, 1.5 MPa). Since a negative peak acoustic pressure of 1.5 MPa is well above the inertial cavitation threshold, these images demonstrate typical US-induced morphological changes, including destruction of the LNP, loss of spherical conformation and daughter LNP formation.

### Healthy hindlimb model

#### Perfusion changes in contrast-enhanced ultrasound imaging

**Figure 4:**
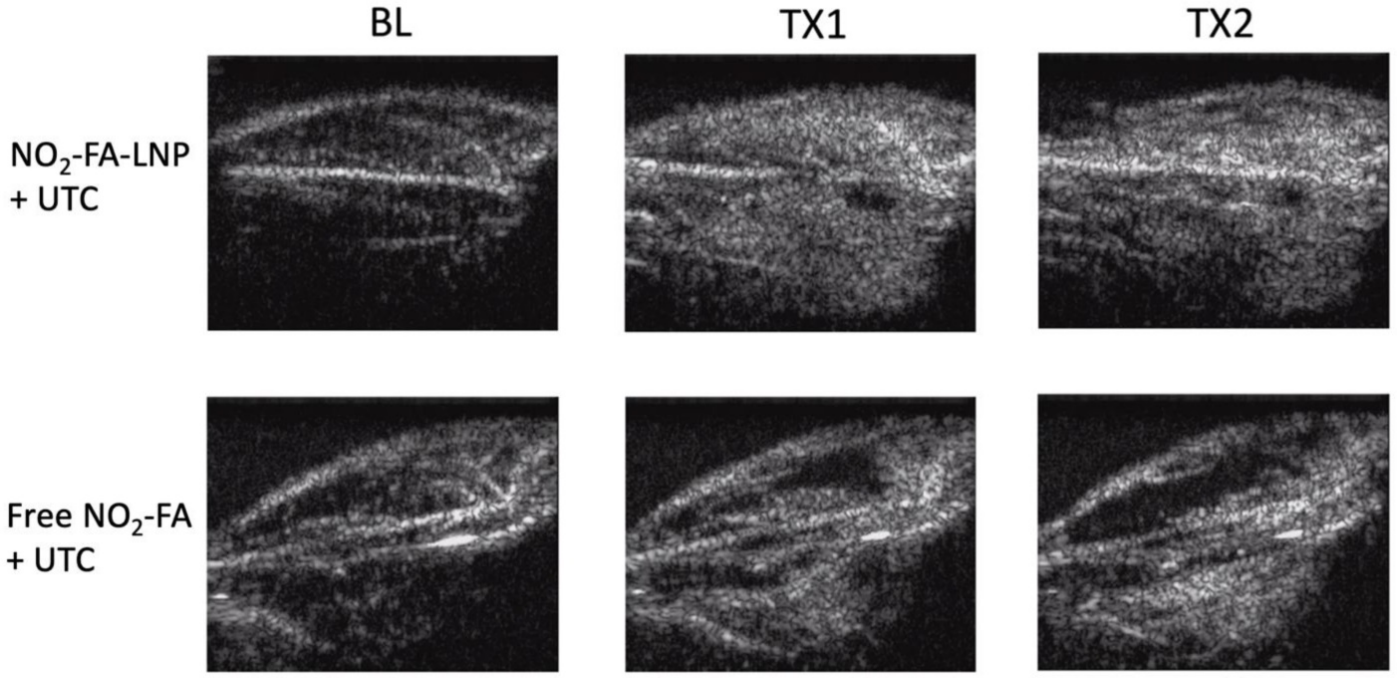
Burst-replenishment still-frame images from contrast-enhanced US imaging of healthy hindlimb model - Still-frame observational analysis at 5 sec post-burst shows drastic differences in replenishment between NO_2_-FA-LNP+UTC and Free NO_2_-FA+UTC groups. Much of the microvascular bed is yet to be replenished in the latter, suggesting possible microvascular slowing or previously discussed microvascular spasm, while the former group shows full replenishment of the microvasculature exceeding baseline levels. TX1 for treatment 1, TX2 for treatment 2 imaging periods after a 10-min treatment.

Burst-replenishment imaging representative still-frame images taken at 5 sec post-burst after each 10 min treatment for two UTC-treated healthy hindlimb groups are shown in Fig. 4. As the NO_2_-FA-LNP group featured direct incorporation of NO_2_-FA into the LNP structure, comparison of these images versus co-infusion with unmodified LNP illustrates the additional effects of NO_2_-FA incorporation over its presence in the vascular compartment.

The NO_2_-FA-LNP group induced a greater area coverage with greater image intensity of contrast-visualized microvascular reperfusion as compared to control conditions. Free NO_2_-FA+UTC does not impact image intensity and reperfusion as compared to baseline at 5 sec post-burst. Although this is not representative of a persistent hypoperfused region given that at 30 sec post-burst, the hypointense region was successfully reperfused, this abridged comparison emphasizes the improved rate of microvascular reperfusion induced by NO_2_-FA-LNP treatment. Still-frame visualization demonstrated increased speed of microvascular perfusion of the NO_2_-FA-LNP group that was further reinforced by quantification of microvascular perfusion using region-of-interest selection.

**Figure 5:**
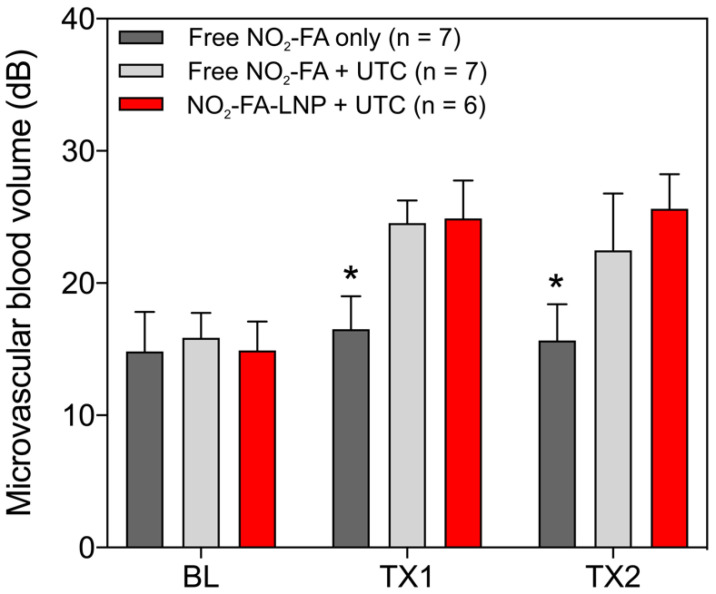
Changes in microvascular blood volume for the healthy hindlimb model - Changes in microvascular blood volume show similarly significant increases from baseline for both NO_2_-FA-LNP+UTC and free NO_2_-FA+UTC groups compared against free NO_2_-FA only. Results were analyzed using two-way ANOVA with post-hoc analysis performed using Sidak's multiple comparisons test. Asterisk indicates *p*<0.05 compared to both other groups. TX1 and TX2 indicate imaging was obtained after each respective 10-min treatment, BL indicates baseline imaging prior to treatments.

**Figure 6:**
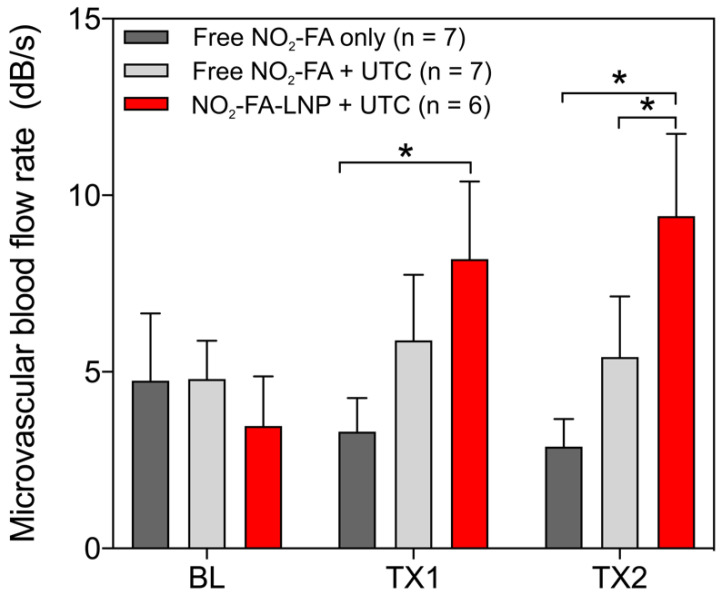
Change in microvascular flow rate for healthy hindlimb model- Only the NO_2_-FA-LNP+UTC group demonstrated significantly increased microvascular blood flow rate compared to free NO_2_-FA only after both treatments. The NO_2_-FA-LNP+UTC group also demonstrated significantly increased microvascular blood flow rate over the free NO_2_-FA+UTC group after treatment 2. Results were analyzed using two-way ANOVA with post-hoc analysis performed using Sidak's multiple comparisons test. Asterisk between bracketed groups indicates *p*<0.05. TX1 and TX2 indicate imaging was obtained after each respective 10-min treatment, BL indicates baseline imaging prior to treatments.

Hindlimb cine-loops were quantified using an exponential regression for region-of-interest image intensity (Fig. 5 and 6). Although both NO_2_-FA-LNP and free NO_2_-FA+UTC groups increased microvascular blood volume, only the NO_2_-FA-LNP group demonstrated increased microvascular flow rate. The administration of free NO_2_-FA did not affect hindlimb microvascular perfusion, revealing that while UTC significantly increased blood volume, the incorporation of NO_2_-FA into LNP was required for enhancement of microvascular perfusion.

#### Tissue delivery of NO_2_-FA

**Figure 7:**
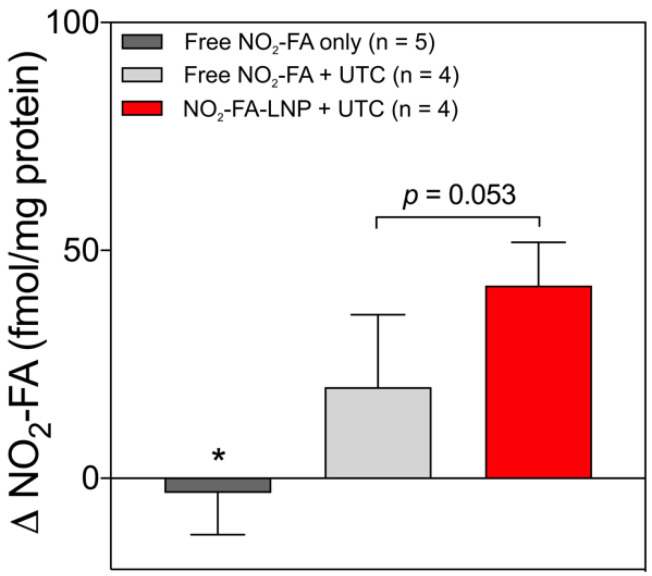
Change in tissue concentration of NO_2_-FA - Changes in concentration were taken as a difference between US treated and untreated regions on the ipsilateral hindlimb on the same animal. All samples were treated as pairwise differences of treated minus untreated concentrations of NO_2_-FA per mg protein. Differences for each group were analyzed using one-way ANOVA with post-hoc analysis using Tukey's multiple comparisons test. Asterisk indicates *p*<0.05 compared against both other groups. Bracketed *p*-value between the two UTC-treated groups is as shown.

Tissue concentrations of NO_2_-FA at both US treated and untreated sites for the three groups are shown (Fig. 7). The administration of free NO_2_-FA showed no difference in tissue concentration when no UTC was applied to the hindlimb. NO_2_-FA co-infusion with UTC and NO_2_-FA-LNP +UTC showed sequentially increasing NO_2_-FA concentration. The prior incorporation of NO_2_-FA into the LNP gave ~2x increase in tissue delivery of NO_2_-FA as compared to NO_2_-FA co-infused with control LNP.

### Ischemia-reperfusion injury model

#### Perfusion changes measured by contrast-enhanced ultrasound imaging

**Figure 8:**
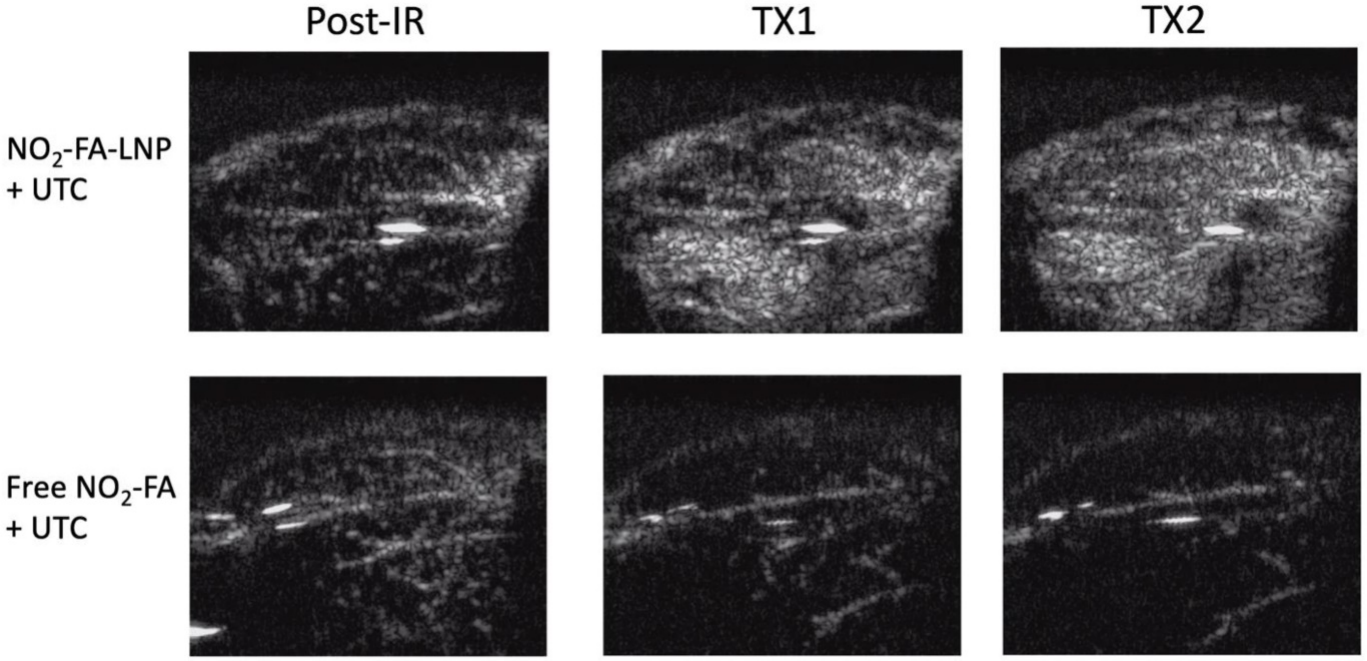
Contrast-enhanced US still-frame images of ischemia-reperfusion injury model - Still-frame images were taken from burst-replenishment contrast-enhanced cine-loops at 5 sec post-burst. Note the complete replenishment of microvasculature in the NO_2_-FA-LNP+UTC group while the free NO_2_-FA+UTC group shows a decrease in microvascular perfusion.

**Figure 9:**
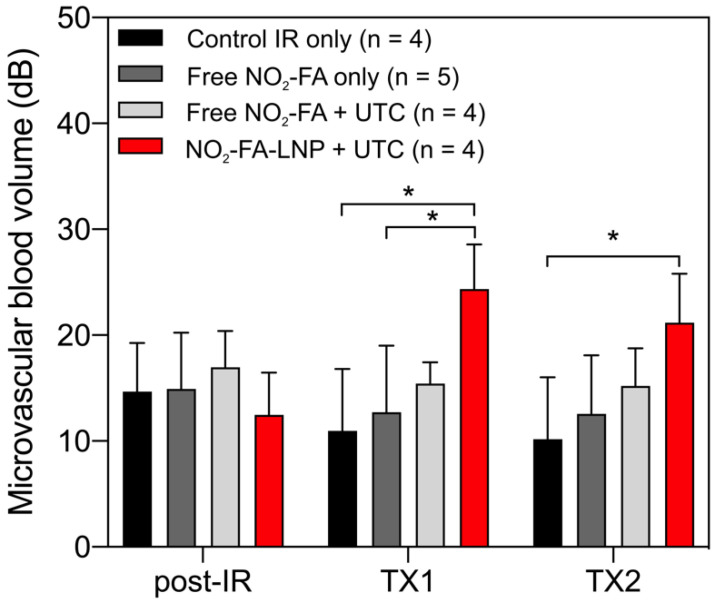
Changes in microvascular blood volume in the IR model - Significant increases in microvascular blood volume are only seen in the NO_2_-FA-LNP+UTC group for the IR model after each treatment. Compared to the healthy hindlimb model, no significant increases in microvascular blood volume were seen in the free NO_2_-FA+UTC group after the post-IR imaging interval. Asterisk indicates *p*<0.05 for bracketed groups. TX1 for treatment 1, TX2 for treatment 2 imaging periods after a 10-min treatment.

**Figure 10:**
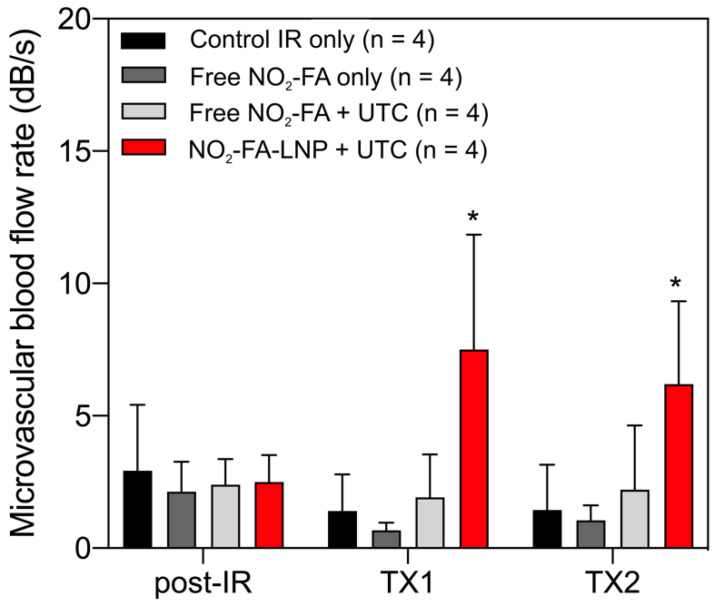
Changes in microvascular blood flow rate in the IR model - As for microvascular blood volume responses, only the NO_2_-FA-LNP+UTC group showed significant increases in microvascular blood flow rate compared to all other groups. Asterisk indicates *p*<0.05 against all other groups. TX1 for treatment 1, TX2 for treatment 2 imaging periods after a 10-min treatment.

Still frames from burst-replenishment cine-loops are shown for the NO_2_-FA-LNP and NO_2_-FA co-infusion with standard UTC groups (Fig. 8). The NO_2_-FA-LNP group showed significant increases in perfusion compared to the post-IR state while the NO_2_-FA co-infusion group showed no improvement in perfusion. Quantitative analysis of hindlimb perfusion showed NO_2_-FA-LNP treatment significantly increased in microvascular blood volume and microvascular blood flow (Fig. 9 and 10).

#### UTC-mediated NO_2_-FA delivery suppresses indices of inflammation in hindlimb subjected to ischemia-reperfusion

**Figure 11:**
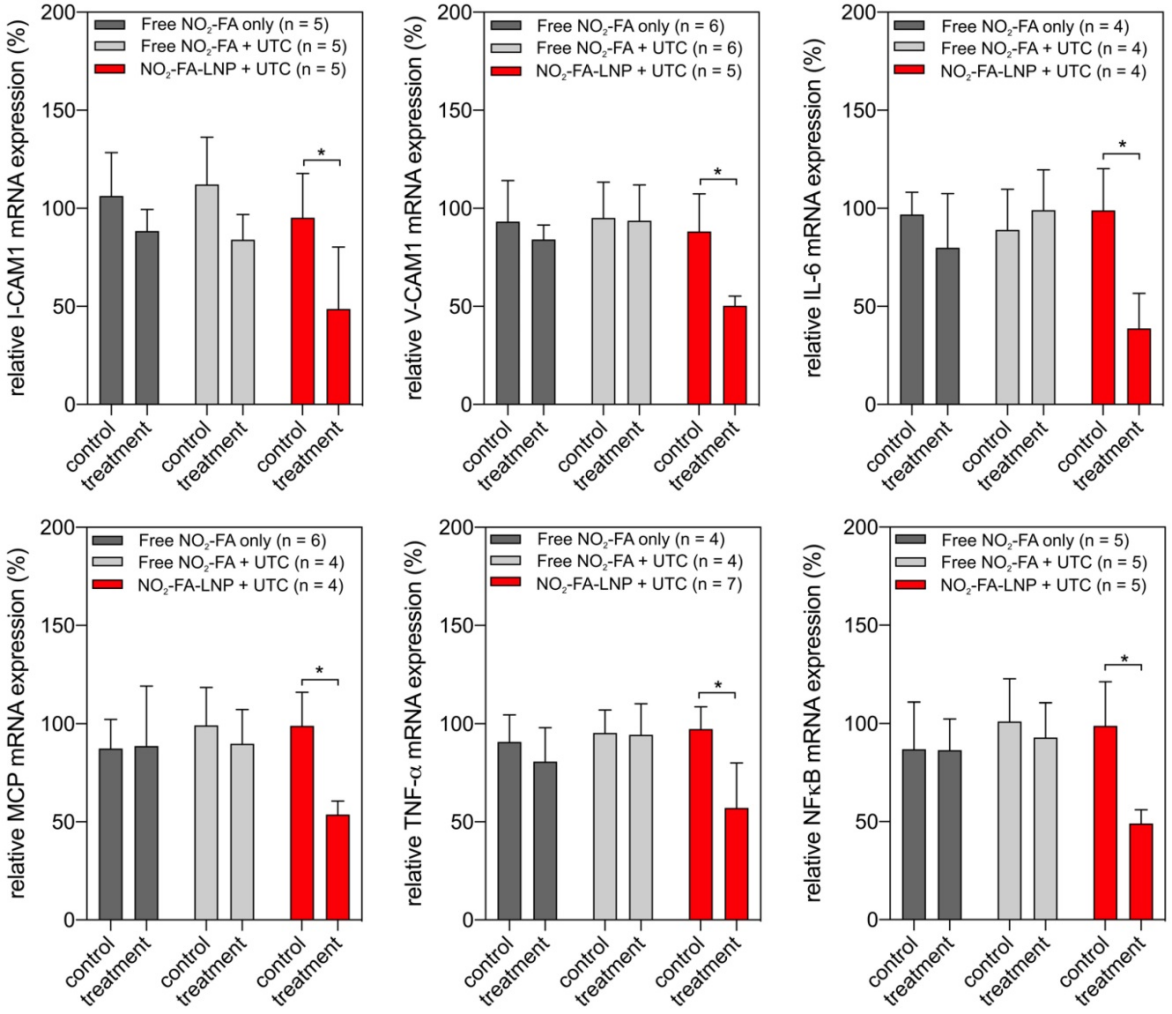
Gene expression changes after NO_2_-FA-LNP-UTC treatment - Changes in gene expression for key genes in inflammation and recruitment of leukocytes are shown for skeletal muscle tissue samples taken from control (no US treatment) and US treated sites on the ipsilateral hindlimb from the same animal. Only the NO_2_-FA-LNP+UTC group showed significant reductions in mRNA expression for the target genes in the treatment site compared to the other groups. All samples were normalized against a control group receiving IR injury only. Control and treatment sites were subjected to the same ischemia-reperfusion injury. Significance was calculated using a 2-way ANOVA with post-hoc comparison using Sidak's multiple comparisons test. Asterisk indicates *p*<0.05 between bracketed groups.

**Figure 12:**
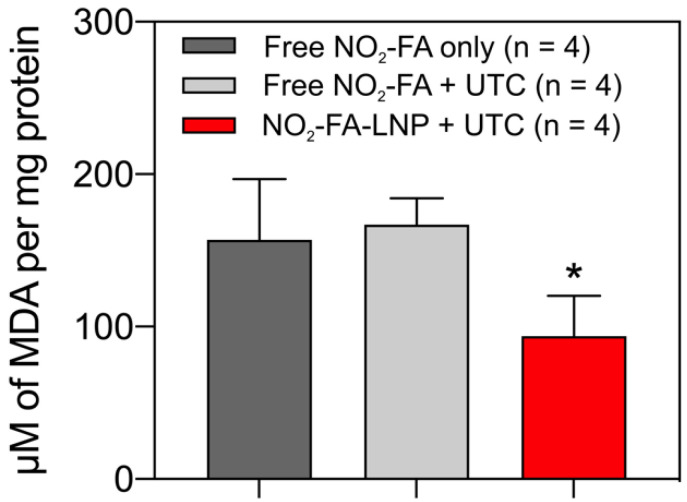
NO_2_-FA-LNP+UTC reduces lipid peroxidation - Measurement of MDA per mg protein for treatment site tissue samples are shown. NO_2_-FA-LNP+UTC resulted in the lowest concentration of MDA indicating the least extent of lipid peroxidation compared to either NO_2_-FA alone or NO_2_-FA+UTC. All samples were normalized against a control group receiving IR injury only. Significance was calculated using a one-way ANOVA with post-hoc analysis using Tukey's multiple comparisons test. Asterisk indicates *p*<0.05 for the indicated group compared to both other groups.

RT-PCR analysis of pro-inflammatory chemokine expression showed UTC-induced NO_2_-FA administration decreased tissue ICAM-1, VCAM-1, MCP-1, and TNF-α mRNA expression (Fig. 11). Lipid oxidation byproduct measurements of TBA-reactive species per mg protein for the three groups revealed that NO_2_-FA-LNP +UTC treatment decreased the generation of TBARS, compared to both free NO_2_-FA and free NO_2_-FA+UTC groups (Fig. 12).

## Discussion

**Figure 13:**
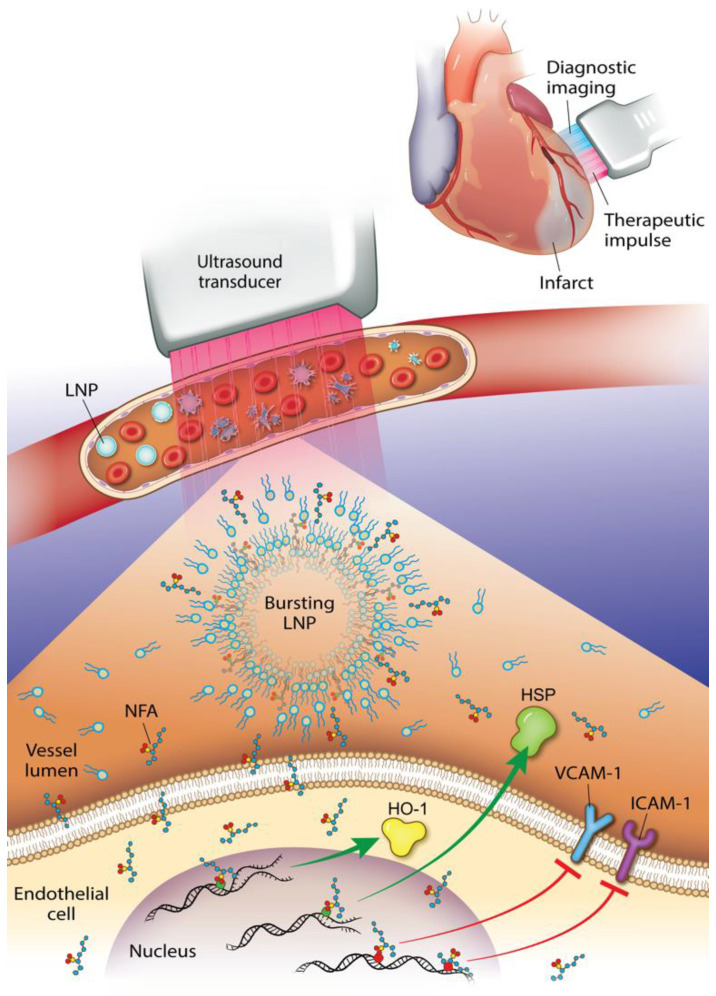
Diagnostic imaging of an infarcted vessel and the treatment of microvascular occlusion via ultrasound-induced focal drug delivery - Lipid nanoparticles (LNP) carrying a nitroalkene fatty acid (NFA) cargo are subjected to ultrasound-induced cavitation that induces LNP breakage and the release of NFA that induce adaptive gene expression and anti-inflammatory responses. This can include up-regulation of tissue-protective heme oxygenase-1 (HO-1) and heat shock proteins (HSP) and the inhibition of pro-inflammatory cytokines and adhesion proteins.

This study reveals that an NO_2_-FA-containing lipid LNP can be locally targeted via image-guided therapeutic US for NO_2_-FA release in the vascular compartment of a rodent hindlimb. These multi-lamellar lipid membranes consisted of di-stearoyl phosphatidylcholine, di-stearoyl phosphatidylethanolamine linked to PEG-2000, polyoxyethylene-40 stearate and having a 22% mol ratio of 10-nitro-octadec-9-enoic acid, the active pharmacological agent. This formulation gave a focal tissue delivery of NO_2_-FA that significantly increased microvascular blood flow and suppressed inflammatory cytokine transcription and oxidative stress following tissue ischemia-reperfusion injury. This affirms that NO_2_-FA can be functionalized in a LNP vehicle to focally deliver a chemically reactive, anti-inflammatory agent when LNP are disrupted by ultra-sound induced cavitation (Fig. 13). This nanotechnology-based drug delivery strategy is novel, since previous preclinical and clinical studies of NO_2_-FA therapeutics have utilized intraperitoneal, sub-cutaneous, intravenous or oral systemic administration routes. Thus, the present approach, can limit off target effects and better treat acutely developing, localized pathologies via the rapid concentration of a therapeutic NO_2_-FA to primary sites of need.

### Chemical and biophysical properties of NO_2_-FA-LNP-delivered active pharmacological ingredient

The fatty acid nitroalkene derivative used herein is a synthetic homolog of endogenously generated mediators that are present in nM concentrations in plants, fish and mammals 22, 59. Unsaturated fatty acids are nitrated by diverse metabolic and inflammatory reactions of NO and nitrite (NO_2_^-^)-derived reactive species that ultimately give rise to the nitrogen dioxide radical (^.^NO_2_). The addition of an electron-withdrawing nitro (R-NO_2_) substituent on a fatty acid double bond confers an electrophilic nature to the β-carbon of the double bond 60. This electron-poor β-carbon is chemically reactive and will undergo a kinetically fast and reversible reaction with electron-rich biological nucleophiles such as the thiolate of cysteine (Cys). This reaction, termed Michael addition, will induce the structural and functional PTM of proteins that have functionally-significant, electrophile-reactive thiols adducted by NO_2_-FA 61.

In pure form NO_2_-FA can self-aggregate into micelles, displaying a critical micellar concentration in aqueous milieu of 5-50 μM 62. The present study affirms that NO_2_-FA stably reside in multilamellar phospholipid membranes as the free acid, where structural integrity and electrophilic chemical reactivity is preserved for days. This unique LNP formulation thus both capitalizes and expands upon on the 25+ yr clinical use of artificial lipid membranes (liposomes, LNP) in nanoscale drug delivery systems. Most recently, the LNP delivery of a mRNA construct has been highly effective for immunizing the population against the SARS-CoV-2 virus. Importantly, this clinical success further affirms the safety, utility and economic viability of using lipid nanoparticles to confer pharmacokinetic advantages to life-saving therapeutic agents via the tissue targeting of labile cargo.

### Mechanisms accounting for increased tissue NO_2_-FA delivery via UTC

When subjected to US-induced cavitation, ultra-high-speed microscopy of NO_2_-FA-LNP revealed acoustic behaviors similar to those of LNP in the absence of 22 mol% NO_2_-FA. This includes inertial cavitation characteristics and the formation of smaller daughter LNP, both viewed as significant to the therapeutic efficacy of lipid LNP in UTC therapy. These properties are also critical in the mechanical “chiseling” of microthrombi when relieving the physical obstruction associated with MVO 63.

The mechanisms underlying the enhanced uptake of the LNP-associated NO_2_-FA cargo can include two physical properties of sonicated LNP, sonoporation and sonoprinting 64. Sonoporation involves the formation of transient micropores in cellular membranes that are induced by LNP cavitational forces tangent to membrane surfaces. Increased endothelial barrier and membrane permeability at cellular junctions has also been reported 65. While sonoporation is an attractive mechanism explaining NO_2_-FA-LNP delivery, the absence of enhanced NO_2_-FA uptake in the free NO_2_-FA+UTC group (utilizing control LNP) argues against sonoporation-promoted tissue uptake.

Sonoprinting involves deposition of LNP components onto cellular membranes as a result of radial oscillatory forces 66. Via this mechanism, the NO_2_-FA cargo can be rapidly trafficked to intracellular targets by associating with the CD36 scavenger receptors on macrophages, the family of fatty acid binding proteins found in all cells and, upon NO_2_-FA-membrane and membrane-membrane contact, via diffusional processes 67, 68, 69. Sonoprinting may thus explain the increased NO_2_-FA uptake for the NO_2_-FA-LNP+UTC group, as opposed to the free NO_2_-FA+UTC group, as incorporation of NO_2_-FA into LNP lamellae was the distinguishing factor between the two. Given that NO_2_-FA must associate with and traverse membranes by diffusional or endocytic mechanisms for gaining access to intracellular compartments such as the cytosol, binding proteins, nuclei and mitochondria, sonoprinting likely facilitates these functions 31.

To date, preclinical and clinical studies of NO_2_-FA therapeutics have utilized systemic delivery strategies. It is shown herein that UTC can rapidly and focally enhance tissue delivery of NO_2_-FA, with two sequential 10 min UTC treatments significantly increasing local skeletal muscle NO_2_-FA concentration well over that of adjacent untreated sites. While the chronic systemic administration of NO_2_-FA has not shown significant safety signals at therapeutic concentrations in humans, the rapid focal delivery of NO_2_-FA remains desirable for instigating protective and adaptive signaling responses in acute and more localized pathologies.

### Anti-inflammatory and adaptive gene expression regulation by NO_2_-FA

The electrophilic character of NO_2_-FA induces reversible Michael addition with Cys thiolates present in proteins and other small molecules occurs. The reaction of NO_2_-FA with specific hyperreactive protein thiols can directly inhibit pro-inflammatory enzymes that have functionally significant Cys moieties. Examples of enzymatic targets in this category include xanthine oxidoreductase, NADPH oxidases, cyclooxygenase and 5-lipoxygenase 70. NO_2_-FA also exert anti-inflammatory responses by modifying transcription factor and signal transduction pathway activities. For example, NO_2_-FA activate the anti-inflammatory transcriptional regulatory proteins Kelch-like ECH-associated protein 1/ nuclear factor (erythroid-derived 2)-like 2 (Keap1/Nrf2) and peroxisome proliferator-activated receptor gamma (PPARγ) 22, 34, 71. NO_2_-FA also inhibit pro-inflammatory signaling responses regulated by Toll-like receptors (TLR), nuclear factor kappa-light-chain-enhancer of activated B cells (NF-κB), janus kinase/signal transducer and activator of transcription proteins (JAK/STAT) and stimulator of interferon genes (STING) 30, 72-74. Via these pleiotropic actions, NO_2_-FA promote an antioxidant and adaptive signaling milieu that limit pathogenic inflammatory cell function, vaso-occlusion and downstream vessel wall remodeling.

These effects were in part evidenced by an inhibition of tissue lipid oxidation by NO_2_-FA-LNP +UTC treatment but not by free NO_2_-FA alone or free NO_2_-FA+UTC. There was also a significant reduction in the expression of numerous inflammatory target genes that are expressed during ischemia-reperfusion injury and contribute to the sequelae of MVO. Specifically, ischemia-reperfusion injury promotes nuclear translocation of NF-κB and upregulates expression of TNF-α, a pro-inflammatory factor secreted by monocytes and vascular endothelial cells. This in turn induces the expression of pro-inflammatory cytokines including IL-6 and MCP-1, as well as adhesion molecules on the surface of vascular endothelial cells such as VCAM-1 and ICAM-1. This cascade is predictive of not only MVO but also future adverse cardiovascular events, as the resulting inflammation produces increased oxidative stress and tissue damage, ultimately worsening patient outcomes 75. There were concurrent decreases in all of these inflammatory indices with NO_2_-FA-LNP+UTC treatment that were not seen in systemic administration of free NO_2_-FA alone or free NO_2_-FA+UTC.

### NO_2_-FA signaling actions promote NO homeostasis and microvascular perfusion

Microvascular hemodynamic changes were assessed herein through contrast-enhanced US imaging. The enhanced local vessel and tissue targeting of NO_2_-FA induced by NO_2_-FA-LNP+UTC can increase NO bioavailability in tissues and the vascular compartment via multiple mechanisms. Notably, NO_2_-FA only show NO donor activities *in vitro* in aqueous phosphate buffer-based studies, an effect mediated via a modified Nef reaction. This NO_2_-FA decay reaction to NO appears to not be operative in more complex biological fluids and tissues where Michael addition is possible and a kinetically more facile reaction 76. In support of this precept, preclinical pharmacokinetics studies and human Phase 1 and Phase 2 trials do not show an acute effect of intravenous or oral NO_2_-FA on blood pressure or pulse rate. This may not rule out other less direct NO and cGMP-dependent signaling mechanisms being enhanced by the local effects of NO_2_-FA-LNP+UTC. For example, NO_2_-FAs also increase eNOS mRNA and protein expression and eNOS serine-1177 phosphorylation. The net effect would be to increase the vascular endothelial caveolar translocation and enzymatic specific activity of eNOS 77.

Given that previous studies of *in vitro* endothelial NO production after NO_2_-FA administration *in vitro* showed significant increases ~2 hr post-treatment, the gene expression responses and post-translational modifications of eNOS that can be induced by NO_2_-FA are less likely the etiology of the acute perfusion changes that were seen herein on the timescale of minutes. Future investigation can identify additional mechanisms responsible for the improved microvascular perfusion induced by NO_2_-FA-LNP+UTC, such as suppression of adhesion molecule expression and other processes of inflammatory cell-mediated vaso-occlusion 30. Despite this unclear etiology, it is apparent that the combination of enhanced perfusion due to the anti-inflammatory and antioxidant effects of NO_2_-FA-LNP+UTC therapy may ameliorate MVO and ischemia-reperfusion injury seen after AMI.

### Cardiovascular impact of NO_2_-FA perturbation of inflammation and metabolism

Previous literature has shown that the inhibitions of various inflammatory signaling reactions by NO2-FA are both time-dependent and dosage-dependent in inflammatory and vascular cells 28. The suppression of vascular inflammatory responses observed with NO_2_-FA-LNP+UTC treatment was consistent with those observed in the more chronic systemic administration of NO_2_-FA 29. This, coupled with previous success in reducing inflammation and myocardial preservation in focal and global ischemia-reperfusion models by NO_2_-FA, suggests the strong potential of further improving therapeutic outcomes by rapidly concentrating NO_2_-FA at the site of the myocardial insult 29. Moreover, hypertensive and oxidative stress-induced cardiac fibrosis and atrial fibrillation were also inhibited by NO_2_-FA administration, further affirming the pleiotropic anti-inflammatory and adaptive signaling actions induced by small molecule electrophiles 35, 38, 39. The present results build upon this body of work by showing that targeted treatment can immediately induce significant changes in microvascular perfusion and gene expression responses can become evident as soon as 2 hr after NO_2_-FA-LNP+UTC treatment.

Limitations: There are limitations to this initial study that has motivated more comprehensive ongoing work. For NO_2_-FA-LNP+UTC therapy, additional observation intervals can be implemented to improve the measurement of short and long-term tissue loading kinetics and extend/expand the characterization of gene expression and hemodynamic parameter changes. Mechanistic analyses identifying the specific source of hemodynamic changes were also not performed; given previous role of NO pathways in UTC therapy, future investigations may include live *in vivo* detection of local changes in NO concentration.

Specific mechanics of LNP interactions with capillary endothelium were not explored in this study. However, our previous studies have shown that the microvascular architecture remains intact after this therapy. Future work may elucidate differences in LNP sizes *in vitro* (Fig. 3) versus *in vivo* in the vasculature through use of high-speed intra-vital microscopy. In addition, contrast-enhanced US is a well-validated technique for measurement of true microvascular perfusion, and the majority of microvascular perfusion is comprised of capillary blood flow. Moreover, improvement in myocardial perfusion, as assessed with contrast-enhanced US, is associated with better clinical outcomes. Analysis of multiple time intervals for NO_2_-FA delivery may also reveal key mechanisms of NO_2_-FA-LNP therapeutic efficacy and improve pharmacokinetic insight.

## Conclusions

The sonolysis of NO_2_-FA-containing LNP revealed therapeutic potential for the acute treatment of vascular occlusion upon tissue ischemia-reperfusion. This rapid, targeted treatment suppressed inflammatory gene expression, limited oxidative stress and improved microvascular perfusion. This novel LNP technology improved the pharmacokinetics of chemically reactive electrophilic lipids when using focal ultrasound as a vehicle for targeted delivery. This therapeutic strategy can be especially suitable for treatment of localized sequelae of AMI, including MVO and ischemia-reperfusion injury, with its focus on concentrating drug payloads at the specific site of MVO. This also provides opportunities for synergy with the other aspects of UTC therapy, including enhanced microvascular perfusion and mechanical disruption of thrombi.

## Non-standard Abbreviations and Acronyms

NO_2_-FA, nitro-fatty acid; LNP, lipid nanoparticle; NO_2_-FA-LNP, nitro-fatty acid lipid nanoparticle; UTC, ultrasound-targeted cavitation of lipid nanoparticles; US, ultrasound; MVO, microvascular obstruction.

## References

[1] Benjamin EJ, Virani SS, Callaway CW, Chamberlain AM, Chang AR, Cheng S (2018). Heart Disease and Stroke Statistics-2018 Update: A Report From the American Heart Association. Circulation.

[2] Smith SC, Feldman TE, Hirshfeld JW, Jacobs AK, Kern MJ, King SB ACC/AHA/SCAI 2005 guideline update for percutaneous coronary intervention: a report of the American College of Cardiology/American Heart Association Task Force on Practice Guidelines (ACC/AHA/SCAI writing committee to update the 2001 guidelines for percutaneous coronary intervention). 2006; 47: e1-e121.

[3] Ibáñez B, Heusch G, Ovize M, Van de Werf FJJotACoC Evolving therapies for myocardial ischemia/reperfusion injury. 2015; 65: 1454-71.

[4] Jaffe R, Dick A, Strauss BH (2010). Prevention and treatment of microvascular obstruction-related myocardial injury and coronary no-reflow following percutaneous coronary intervention: a systematic approach. JACC Cardiovasc Interv.

[5] Gupta S, Gupta MM (2016). No reflow phenomenon in percutaneous coronary interventions in ST-segment elevation myocardial infarction. Indian Heart J.

[6] Niccoli G, Burzotta F, Galiuto L, Crea F (2009). Myocardial no-reflow in humans. J Am Coll Cardiol.

[7] Kaul S (2014). The "no reflow" phenomenon following acute myocardial infarction: mechanisms and treatment options. J Cardiol.

[8] Bekkers SC, Yazdani SK, Virmani R, Waltenberger J (2010). Microvascular obstruction: underlying pathophysiology and clinical diagnosis. J Am Coll Cardiol.

[9] Reffelmann T, Kloner RAJBric The no-reflow phenomenon: a basic mechanism of myocardial ischemia and reperfusion. 2006; 101: 359-72.

[10] Jaffe R, Charron T, Puley G, Dick A, Strauss BH (2008). Microvascular obstruction and the no-reflow phenomenon after percutaneous coronary intervention. Circulation.

[11] Galli A, Lombardi F (2016). Postinfarct Left Ventricular Remodelling: A Prevailing Cause of Heart Failure. Cardiol Res Pract.

[12] Mazhar J, Mashicharan M, Farshid A (2016). Predictors and outcome of no-reflow post primary percutaneous coronary intervention for ST elevation myocardial infarction. Int J Cardiol Heart Vasc.

[13] Bouleti C, Mewton N, Germain S (2015). The no-reflow phenomenon: State of the art. Arch Cardiovasc Dis.

[14] Hamirani YS, Wong A, Kramer CM, Salerno M (2014). Effect of microvascular obstruction and intramyocardial hemorrhage by CMR on LV remodeling and outcomes after myocardial infarction: a systematic review and meta-analysis. JACC Cardiovasc Imaging.

[15] Ndrepepa G, Tiroch K, Fusaro M, Keta D, Seyfarth M, Byrne RA 5-year prognostic value of no-reflow phenomenon after percutaneous coronary intervention in patients with acute myocardial infarction. 2010; 55: 2383-9.

[16] Khorramirouz R, Corban MT, Yang SW, Lewis BR, Bois J, Foley T (2018). Microvascular obstruction in non-infarct related coronary arteries is an independent predictor of major adverse cardiovascular events in patients with ST segment-elevation myocardial infarction. Int J Cardiol.

[17] de Waha S, Patel MR, Granger CB, Ohman EM, Maehara A, Eitel I (2017). Relationship between microvascular obstruction and adverse events following primary percutaneous coronary intervention for ST-segment elevation myocardial infarction: an individual patient data pooled analysis from seven randomized trials. Eur Heart J.

[18] Rezkalla SH, Stankowski RV, Hanna J, Kloner RA (2017). Management of No-Reflow Phenomenon in the Catheterization Laboratory. JACC Cardiovasc Interv.

[19] Fiotti N, Giansante C, Ponte E, Delbello C, Calabrese S, Zacchi T (1999). Atherosclerosis and inflammation. Patterns of cytokine regulation in patients with peripheral arterial disease. Atherosclerosis.

[20] Hartge MM, Unger T, Kintscher U (2007). The endothelium and vascular inflammation in diabetes. Diabetes and Vascular Disease Research.

[21] Lejay A, Fang F, John R, Van JA, Barr M, Thaveau F (2016). Ischemia reperfusion injury, ischemic conditioning and diabetes mellitus. Journal of Molecular and Cellular Cardiology.

[22] Freeman BA, O'Donnell VB, Schopfer FJ (2018). The discovery of nitro-fatty acids as products of metabolic and inflammatory reactions and mediators of adaptive cell signaling. Nitric oxide: biology and chemistry.

[23] Zhao Y, Chang Z, Zhao G, Lu H, Xiong W, Liang W (2020). Suppression of Vascular Macrophage Activation by Nitro-Oleic Acid and its Implication for Abdominal Aortic Aneurysm Therapy. Cardiovasc Drugs Ther.

[24] Schopfer FJ, Khoo NKH (2019). Nitro-Fatty Acid Logistics: Formation, Biodistribution, Signaling, and Pharmacology. Trends Endocrinol Metab.

[25] Lu H, Sun J, Liang W, Zhang J, Rom O, Garcia-Barrio MT (2019). Novel gene regulatory networks identified in response to nitro-conjugated linoleic acid in human endothelial cells. Physiological genomics.

[26] Go YM, Chandler JD, Jones DP (2015). The cysteine proteome. Free radical biology & medicine.

[27] Rubbo H (2013). Nitro-fatty acids: novel anti-inflammatory lipid mediators. Braz J Med Biol Res.

[28] Villacorta L, Gao Z, Schopfer FJ, Freeman BA, Chen YE (2016). Nitro-fatty acids in cardiovascular regulation and diseases: characteristics and molecular mechanisms. Frontiers in bioscience (Landmark edition).

[29] Rudolph V, Rudolph TK, Schopfer FJ, Bonacci G, Woodcock SR, Cole MP (2010). Endogenous generation and protective effects of nitro-fatty acids in a murine model of focal cardiac ischaemia and reperfusion. Cardiovasc Res.

[30] Cui T, Schopfer FJ, Zhang J, Chen K, Ichikawa T, Baker PR (2006). Nitrated fatty acids: Endogenous anti-inflammatory signaling mediators. The Journal of biological chemistry.

[31] Freeman BA, Baker PR, Schopfer FJ, Woodcock SR, Napolitano A, d'Ischia M (2008). Nitro-fatty acid formation and signaling. J Biol Chem.

[32] Kansanen E, Jyrkkanen HK, Volger OL, Leinonen H, Kivela AM, Hakkinen SK (2009). Nrf2-dependent and -independent responses to nitro-fatty acids in human endothelial cells: identification of heat shock response as the major pathway activated by nitro-oleic acid. J Biol Chem.

[33] Baker PR, Lin Y, Schopfer FJ, Woodcock SR, Groeger AL, Batthyany C (2005). Fatty acid transduction of nitric oxide signaling: multiple nitrated unsaturated fatty acid derivatives exist in human blood and urine and serve as endogenous peroxisome proliferator-activated receptor ligands. The Journal of biological chemistry.

[34] Schopfer FJ, Lin Y, Baker PR, Cui T, Garcia-Barrio M, Zhang J (2005). Nitrolinoleic acid: an endogenous peroxisome proliferator-activated receptor gamma ligand. Proceedings of the National Academy of Sciences of the United States of America.

[35] Cole MP, Rudolph TK, Khoo NK, Motanya UN, Golin-Bisello F, Wertz JW (2009). Nitro-fatty acid inhibition of neointima formation after endoluminal vessel injury. Circ Res.

[36] Koudelka A, Ambrozova G, Klinke A, Fidlerova T, Martiskova H, Kuchta R (2016). Nitro-Oleic Acid Prevents Hypoxia- and Asymmetric Dimethylarginine-Induced Pulmonary Endothelial Dysfunction. Cardiovasc Drugs Ther.

[37] Ambrozova G, Martiskova H, Koudelka A, Ravekes T, Rudolph TK, Klinke A (2016). Nitro-oleic acid modulates classical and regulatory activation of macrophages and their involvement in pro-fibrotic responses. Free Radic Biol Med.

[38] Rudolph TK, Ravekes T, Klinke A, Friedrichs K, Mollenhauer M, Pekarova M (2016). Nitrated fatty acids suppress angiotensin II-mediated fibrotic remodelling and atrial fibrillation. Cardiovasc Res.

[39] Mollenhauer M, Mehrkens D, Rudolph V (2018). Nitrated fatty acids in cardiovascular diseases. Nitric Oxide.

[40] Nadtochiy SM, Zhu QM, Urciuoli W, Rafikov R, Black SM, Brookes PS (2012). Nitroalkenes confer acute cardioprotection via adenine nucleotide translocase 1. J Biol Chem.

[41] Nadtochiy SM, Madukwe J, Hagen F, Brookes PS (2014). Mitochondrially targeted nitro-linoleate: a new tool for the study of cardioprotection. Br J Pharmacol.

[42] Nadtochiy SM, Baker PR, Freeman BA, Brookes PS (2009). Mitochondrial nitroalkene formation and mild uncoupling in ischaemic preconditioning: implications for cardioprotection. Cardiovasc Res.

[43] Kelley EE (2019). Diminishing Inflammation by Reducing Oxidant Generation: Nitrated Fatty Acid-Mediated Inactivation of Xanthine Oxidoreductase. Bioactive Lipids in Health and Disease.

[44] Kelley EE, Batthyany CI, Hundley NJ, Woodcock SR, Bonacci G, Del Rio JM (2008). Nitro-oleic acid, a novel and irreversible inhibitor of xanthine oxidoreductase. Journal of Biological Chemistry.

[45] Klinke A, Möller A, Pekarova M, Ravekes T, Friedrichs K, Berlin M (2014). Protective effects of 10-nitro-oleic acid in a hypoxia-induced murine model of pulmonary hypertension. American journal of respiratory cell and molecular biology.

[46] Schopfer FJ, Vitturi DA, Jorkasky DK, Freeman BA (2018). Nitro-fatty acids: new drug candidates for chronic inflammatory and fibrotic diseases. Nitric Oxide.

[47] Pacella JJ, Brands J, Schnatz FG, Black JJ, Chen X, Villanueva FSJUim Treatment of microvascular micro-embolization using microbubbles and long-tone-burst ultrasound: an in vivo study. 2015; 41: 456-64.

[48] Yu FT, Chen X, Straub AC, Pacella JJ (2017). The Role of Nitric Oxide during Sonoreperfusion of Microvascular Obstruction. Theranostics.

[49] Leeman JE, Kim JS, Francois T, Chen X, Kim K, Wang J (2012). Effect of acoustic conditions on microbubble-mediated microvascular sonothrombolysis. Ultrasound in Medicine and Biology.

[50] Chen X, Leeman JE, Wang J, Pacella JJ, Villanueva FSJUim, biology New insights into mechanisms of sonothrombolysis using ultra-high-speed imaging. 2014; 40: 258-62.

[51] Carson AR, McTiernan CF, Lavery L, Hodnick A, Grata M, Leng X Gene therapy of carcinoma using ultrasound-targeted microbubble destruction. 2011; 37: 393-402.

[52] Carson AR, McTiernan CF, Lavery L, Grata M, Leng X, Wang J Ultrasound-targeted microbubble destruction to deliver siRNA cancer therapy. 2012.

[53] Kopechek JA, Carson AR, McTiernan CF, Chen X, Hasjim B, Lavery L Ultrasound targeted microbubble destruction-mediated delivery of a transcription factor decoy inhibits STAT3 signaling and tumor growth. 2015; 5: 1378.

[54] Kopechek JA, Carson AR, McTiernan CF, Chen X, Klein EC, Villanueva FSJPo Cardiac gene expression knockdown using small inhibitory RNA-loaded microbubbles and ultrasound. 2016; 11: e0159751.

[55] Yu FT, Chen X, Wang J, Qin B, Villanueva FS (2016). Low Intensity Ultrasound Mediated Liposomal Doxorubicin Delivery Using Polymer Microbubbles. Mol Pharm.

[56] Yu GZ, Istvanic F, Chen X, Nouraie M, Shiva S, Straub AC (2020). Ultrasound-Targeted Microbubble Cavitation with Sodium Nitrite Synergistically Enhances Nitric Oxide Production and Microvascular Perfusion. Ultrasound Med Biol.

[57] Istvanic F, Gary ZY, Francois T, Powers J, Chen X, Pacella JJ (2020). Sonoreperfusion therapy for microvascular obstruction: A step toward clinical translation. Ultrasound in Medicine & Biology.

[58] Yu FTH, Chen X, Straub AC, Pacella JJ (2017). The Role of Nitric Oxide during Sonoreperfusion of Microvascular Obstruction. Theranostics.

[59] Delmastro-Greenwood M, Hughan KS, Vitturi DA, Salvatore SR, Grimes G, Potti G (2015). Nitrite and nitrate-dependent generation of anti-inflammatory fatty acid nitroalkenes. Free Radical Biology and Medicine.

[60] Vitturi DA, Minarrieta L, Salvatore SR, Postlethwait EM, Fazzari M, Ferrer-Sueta G (2015). Convergence of biological nitration and nitrosation via symmetrical nitrous anhydride. Nature chemical biology.

[61] Delmastro-Greenwood M, Freeman BA, Wendell SG (2014). Redox-dependent anti-inflammatory signaling actions of unsaturated fatty acids. Annual review of physiology.

[62] Grippo V, Mojovic M, Pavicevic A, Kabelac M, Hubatka F, Turanek J (2020). Electrophilic characteristics and aqueous behavior of fatty acid nitroalkenes. Redox biology.

[63] Chen X, Wang J, Pacella JJ, Villanueva FSJUim, biology Dynamic behavior of microbubbles during long ultrasound tone-burst excitation: Mechanistic insights into ultrasound-microbubble mediated therapeutics using high-speed imaging and cavitation detection. 2016; 42: 528-38.

[64] Lentacker I, De Cock I, Deckers R, De Smedt S, Moonen CJAddr Understanding ultrasound induced sonoporation: definitions and underlying mechanisms. 2014; 72: 49-64.

[65] Helfield B, Chen X, Watkins SC, Villanueva FS (2016). Biophysical insight into mechanisms of sonoporation. Proc Natl Acad Sci U S A.

[66] De Cock I, Lajoinie G, Versluis M, De Smedt SC, Lentacker IJB Sonoprinting and the importance of microbubble loading for the ultrasound mediated cellular delivery of nanoparticles. 2016; 83: 294-307.

[67] Vazquez MM, Gutierrez MV, Salvatore SR, Puiatti M, Dato VA, Chiabrando GA (2020). Nitro-oleic acid, a ligand of CD36, reduces cholesterol accumulation by modulating oxidized-LDL uptake and cholesterol efflux in RAW264.7 macrophages. Redox biology.

[68] Lamas Bervejillo M, Bonanata J, Franchini GR, Richeri A, Marques JM, Freeman BA (2020). A FABP4-PPARgamma signaling axis regulates human monocyte responses to electrophilic fatty acid nitroalkenes. Redox biology.

[69] Jackson Catherine L, Walch L, Verbavatz J-M (2016). Lipids and Their Trafficking: An Integral Part of Cellular Organization. Developmental cell.

[70] Freeman BA, Pekarova M, Rubbo H, Trostchansky A (2017). Electrophilic nitro-fatty acids: Nitric oxide and nitrite-derived metabolic and inflammatory signaling mediators. In: Ignarro LJ, Freeman BA, editors. Nitric Oxide: Biology and Pathobiology, Third Edition. London, England: Elsevier.

[71] Villacorta L, Minarrieta L, Salvatore SR, Khoo NK, Rom O, Gao Z (2018). In situ generation, metabolism and immunomodulatory signaling actions of nitro-conjugated linoleic acid in a murine model of inflammation. Redox biology.

[72] Villacorta L, Chang L, Salvatore SR, Ichikawa T, Zhang J, Petrovic-Djergovic D (2013). Electrophilic nitro-fatty acids inhibit vascular inflammation by disrupting LPS-dependent TLR4 signalling in lipid rafts. Cardiovasc Res.

[73] Ichikawa T, Zhang J, Chen K, Liu Y, Schopfer FJ, Baker PR (2008). Nitroalkenes suppress lipopolysaccharide-induced signal transducer and activator of transcription signaling in macrophages: a critical role of mitogen-activated protein kinase phosphatase 1. Endocrinology.

[74] Hansen AL, Buchan GJ, Ruhl M, Mukai K, Salvatore SR, Ogawa E (2018). Nitro-fatty acids are formed in response to virus infection and are potent inhibitors of STING palmitoylation and signaling. Proceedings of the National Academy of Sciences of the United States of America.

[75] Yellon DM, Hausenloy DJ (2007). Myocardial reperfusion injury. New England Journal of Medicine.

[76] Schopfer FJ, Baker PRS, Giles G, Chumley P, Batthyany C, Crawford J (2005). Fatty acid transduction of nitric oxide signaling: Nitrolinoleic acid is a hydrophobically stabilized nitric oxide donor. J Biol Chem.

[77] Khoo NK, Rudolph V, Cole MP, Golin-Bisello F, Schopfer FJ, Woodcock SR (2010). Activation of vascular endothelial nitric oxide synthase and heme oxygenase-1 expression by electrophilic nitro-fatty acids. Free Radic Biol Med.

